# Detection and quantification of extracellular microRNAs in murine biofluids

**DOI:** 10.1186/1480-9222-16-5

**Published:** 2014-03-14

**Authors:** Thomas C Roberts, Anna M L Coenen-Stass, Corinne A Betts, Matthew J A Wood

**Affiliations:** 1 Department of Physiology, Anatomy and Genetics, University of Oxford, South Parks Road, OX1 3QX Oxford, UK; 2 Department of Molecular and Experimental Medicine, The Scripps Research Institute, 10550 N. Torrey Pines Road, 92037 La Jolla, CA, USA

**Keywords:** Extracellular microRNA, miRNA, Biofluid, RT-qPCR, Serum, Plasma

## Abstract

**Background:**

MicroRNAs (miRNAs) are short RNA molecules which regulate gene expression in eukaryotic cells, and are abundant and stable in biofluids such as blood serum and plasma. As such, there has been heightened interest in the utility of extracellular miRNAs as minimally invasive biomarkers for diagnosis and monitoring of a wide range of human pathologies. However, quantification of extracellular miRNAs is subject to a number of specific challenges, including the relatively low RNA content of biofluids, the possibility of contamination with serum proteins (including RNases and PCR inhibitors), hemolysis, platelet contamination/activation, a lack of well-established reference miRNAs and the biochemical properties of miRNAs themselves. Protocols for the detection and quantification of miRNAs in biofluids are therefore of high interest.

**Results:**

The following protocol was validated by quantifying miRNA abundance in C57 (wild-type) and dystrophin-deficient (*mdx*) mice. Important differences in miRNA abundance were observed depending on whether blood was taken from the jugular or tail vein. Furthermore, efficiency of miRNA recovery was reduced when sample volumes greater than 50 μl were used.

**Conclusions:**

Here we describe robust and novel procedures to harvest murine serum/plasma, extract biofluid RNA, amplify specific miRNAs by RT-qPCR and analyze the resulting data, enabling the determination of relative and absolute miRNA abundance in extracellular biofluids with high accuracy, specificity and sensitivity.

## Background

MicroRNAs (miRNAs) are short RNA molecules involved in gene regulation in higher organisms [1] which have recently been detected in extracellular biofluids including serum/plasma [2,3], urine [4], cerebral spinal fluid [5], saliva [6] and seminal fluid [7]. Since their discovery in 1993 [8] miRNAs have been the subject of intense study on account of their involvement in a plethora of physiological and pathophysiological processes. The presence of miRNAs in biofluids was somewhat surprising, as the extracellular space is an RNase-rich environment where synthetic RNA oligonucleotides are rapidly degraded (within seconds) after systemic injection [9]. It is now known that miRNAs are protected from nucleolytic degradation by encapsulation within extracellular vesicles [10-12], or by forming complexes with proteins and/or lipoproteins [13-15]. Given that miRNAs are abundant and highly stable in biofluids they have attracted much interest as potential biomarkers for human disease, particularly in the context of cancer [2,3], but also for neurological conditions [16], diabetes [17], hepatitis [18], atherosclerosis [19], preeclampsia [20], and kidney disease [21], to name just a few. Considering the role of miRNAs in the control of gene expression, and their tissue-specific expression [22], their detection in the extracellular space can serve as a minimally-invasive ‘snapshot’ of underlying gene regulation, and therefore physiology and pathophysiology, in their tissues of origin. Whether or not extracellular miRNAs can be taken up by tissues, and thereby transfer gene regulatory information between cells, is currently a matter for debate and an exciting area of ongoing research [10,11,23,24].

The study of extracellular miRNAs in the context of the inherited muscle-wasting disorder Duchenne muscular dystrophy (DMD) is of particular interest. The absence of dystrophin protein in the muscles of DMD patients sensitizes myofibres to contractile damage, leading to inflammation, necrosis, chronic cycles of degeneration and regeneration and ultimately to depletion of the muscle stem cell pool [25]. At present, there are no effective treatments available, although a number of experimental therapies have shown promise in clinical trials [26,27]. Consequently, there is an urgent need to develop minimally-invasive biomarkers for monitoring both disease progression and the efficacy of potential therapeutics. A well-described set of miRNAs (i.e. miR-1, miR-133 and miR-206) is elevated in the circulation of DMD patients and dystrophic animal models [28-33]. Importantly, these miRNAs are primarily expressed in muscle, and regulate myogenic differentiation [34,35], and their abundance correlates with the progression of dystrophic pathology [31] suggesting that their measurement in serum can provide information about the physiology of the muscles from which they originate. The muscle-specificity of these miRNAs means that their measurement is less susceptible to potentially confounding variables such as hemolysis [36] and platelet activation/contamination [37,38] than many other miRNAs. Furthermore, large changes in miRNA abundance are typically observed when comparing dystrophic versus unaffected patients/animals (~50-100 fold change), which is likely a consequence of body-wide release of miRNAs from the musculature. In contrast, detecting a specific miRNA signal from tumors may be more difficult on account of their relatively tiny mass [24]. The combination of these factors makes animal models of DMD ideal systems for the investigation of disease biomarkers and extracellular miRNA biology, and for the development and optimization of novel methods for their measurement.

The detection and quantification of miRNAs in the extracellular space is subject to a number of specific challenges. Firstly, the quality of biofluid samples may be compromised by hemolysis or platelet activation/contamination. Biofluids typically contain high concentrations of proteins (including RNases and PCR inhibitors) and low amounts of RNA (leading to variable RNA extraction efficiencies). Furthermore, the amplification of miRNA sequences requires special detection technologies. The strict constraints on primer design, and the co-purification of PCR inhibitors, may result in sub-optimal PCR efficiencies which must be taken into account for accurate miRNA quantification. Lastly, there are no established reference miRNAs for biofluids, which complicates normalization of miRNA expression.

The protocol described here focuses on the detection and quantification of extracellular miRNAs in blood serum and plasma, but is equally applicable to other biofluids. For example, detection of miRNAs in urine and saliva may be preferable to serum or plasma as collection of these biofluids requires no invasive procedures. Conversely, cerebral spinal fluid (CSF) is of particular interest in the context of neurological disorders due to its anatomical proximity to the brain and spinal cord. For example, the miRNA let-7b is present at high levels in the CSF of Alzheimer’s disease and promotes neurodegeneration via the stimulation of TLR7 [39]. Importantly, the described procedures may also be applied to biofluids from non-murine organisms such as humans. Similarly, the protocol can easily be adapted to detect other small extracellular RNA species such as transfer RNAs, small nuclear RNAs, small nucleolar RNAs and PIWI-interacting RNAs which may have utility as disease biomarkers in other specific circumstances. Small interfering RNAs (siRNAs) can also be detected using the described methodologies (both endogenous-siRNAs and exogenous therapeutic siRNA oligonucleotides). As interest in the extracellular RNA field grows, demand for a protocol for quantification of these species will become more desirable and important. Additionally, with minor modification this protocol can be adapted to detect long RNA transcripts such as messenger RNAs, long non-coding RNAs and virus-derived RNAs, and cell-free DNA which are all known to be present in the extracellular space. The biofluid collection and RNA extraction components of the protocol are also suitable for preparing RNA for next generation sequencing methodologies such as small RNA-seq.

**Figure 1:**
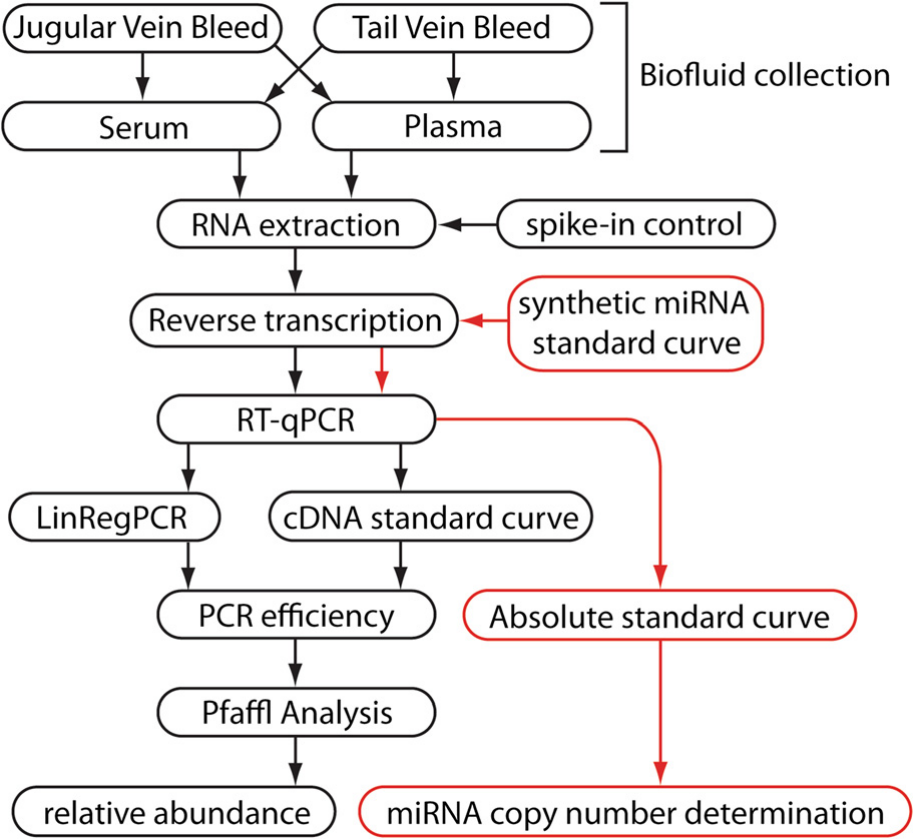
Flowchart depicting the stages of miRNA quantification from murine biofluids.

This protocol describes the collection of murine serum and plasma, extraction of biofluid RNA, miRNA quantification by Reverse Transcriptase-quantitative Polymerase Chain Reaction (RT-qPCR) and downstream data analysis (Figure 1). miRNA abundance can be determined from as little as 10 μl of biofluid (although 50 μl is optimal).

Both serum and plasma samples are used for clinical biochemistry analyses (with serum being used in the majority of cases). Plasma is the cell-free, liquid component of blood. Serum is plasma which has been allowed to clot and is therefore depleted of clotting-associated proteins (e.g. fibrinogen, fibrin etc.). As the clotting process occurs spontaneously following collection, clinical plasma collections are treated with anticoagulant additives. One such additive is sodium/lithium heparin which has been shown to be a potent PCR inhibitor. Heparinized plasma is therefore not suitable for the analysis of extracellular miRNAs. On the other hand, plasma-EDTA is not subject to this limitation and presents an advantage over serum as clotting-induced platelet activation is avoided. Two methods of biofluid harvesting are considered here (a) jugular vein bleed, and (b) tail vein bleed. Generally, a greater volume of blood can be obtained from the jugular vein, although the procedure is terminal. Conversely, tail vein bleeds provide less blood but enable serial measurement of extracellular miRNAs over time in the same mouse. In cases where large changes in serum miRNAs are observed (i.e. 50-100 fold) 3 biological replicates are sufficient to detect a statistically significant difference. However, in other biological contexts the number of replicates required for sufficient statistical power must be formally determined by power analysis.

Extraction of RNA from biofluid samples is subject to technical problems not associated with extraction from tissues or cells. Serum samples contain high concentrations of serum proteins (e.g. albumin). As a result, serum proteins that co-purify with RNA during nucleic acid extraction include RNases, which reduce RNA yield and quality, and inhibitors of downstream enzymatic steps (i.e. reverse transcription and qPCR). Conversely, biofluids typically contain relatively low amounts of nucleic acid and so maximizing RNA yield and ensuring uniform extraction efficiencies are of paramount importance. Low RNA yields can result in failure to detect miRNAs, whereas inconsistent extraction efficiencies can introduce systematic error in miRNA quantification.

We have successfully utilized guanidinium thiocyanate acid phenol chloroform-based methods (i.e. TRIzol LS) [40] for serum RNA extraction. Nucleic acids are separated by organic/aqueous phase separation and isopropanol precipitation. A number of measures can be taken to maximize RNA yield. For example, RNase-free glycogen can be added at the isopropanol precipitation stage which acts as a nucleic acid carrier and makes visualization of the RNA pellet much easier. Similarly, carrier RNA (e.g. bacteriophage MS2 RNA) has also been used to maximize RNA recovery [41]. For a comparison of biofluid RNA extraction methods see reference [5]. The use of an external spike-in control oligonucleotide added at the phenolic extraction phase can be used to monitor the efficiency of extraction. Variation in the abundance of the spike-in control provides an estimate of the variation in extraction efficiency between samples. Mock extractions of water ‘blank’ samples, to which the spike-in control has also been added, can be used to monitor PCR inhibition. (Similar amplification of the spike-in oligonucleotide between experimental samples and blank controls indicates that PCR inhibitors have not been co-purified with biofluid RNA). Using the protocol described here, we have observed little variation in extraction efficiency between samples [42] and evidence of minimal co-purification of PCR inhibitors [31].

Hemolysis, which is the rupture of erythrocytes and release of their cellular contents into the circulation, has the potential to confound extracellular miRNA analysis. Hemolysis can be assessed by visual inspection or spectrophotometry at 540 nm. Alternatively, hemolysis can more quantitatively be assessed by measuring the ratio of miR-451 (an erythrocyte-enriched miRNA) to miR-23a (a highly expressed and stable serum miRNA) abundance. Samples in which the miR-451/miR-23a ratio exceeds 8 are considered hemolysed [41]. Similarly, a recent report showed that quantification of miRNAs can be affected by platelet contamination [37]. As a result, clinical hematological analyses (i.e. full blood count) can be performed in parallel to miRNA analysis in order to quantify platelet contamination.

**Figure 2:**
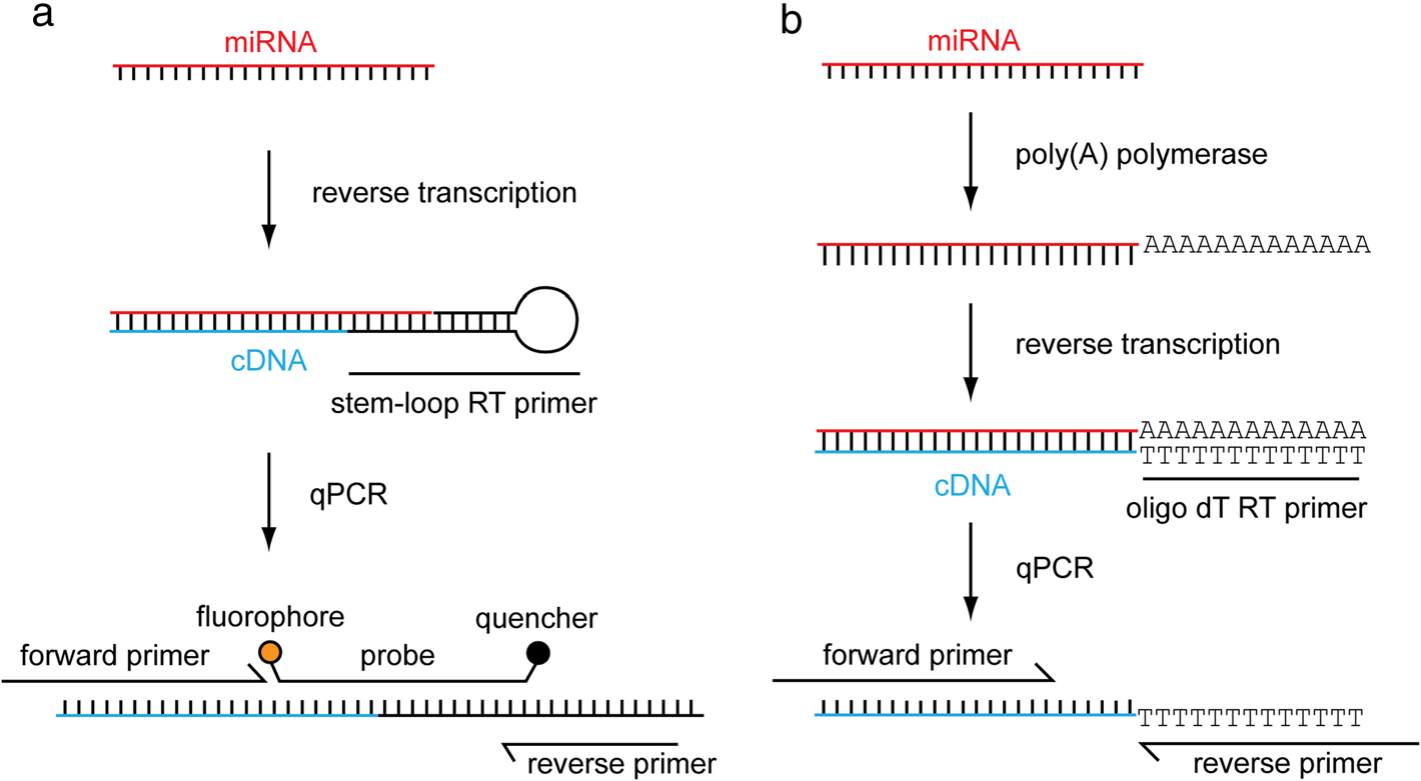
**Methods of miRNA detection and quantification by RT-qPCR. (a)** A single miRNA is reverse transcribed using a gene-specific stem-loop primer. The resulting cDNA is then amplified using a cocktail of specific primers and a hydrolyzable probe. Procession of the *Taq* polymerase displaces, and hydrolyzes, the probe resulting in separation of the fluorophore and quencher. Accumulation of probe fluorescence signal is used to monitor the PCR reaction in real-time. **(b)** Alternatively all miRNAs are polyadenylated by poly(A) polymerase. Tailed miRNAs are then reverse transcribed using an oligo dT priming strategy. The resulting cDNA is amplified using specific primers (often containing LNA nucleotides to increase the primer T_m_). The PCR reaction is monitored in real-time using a dye that fluoresces when bound to double-stranded DNA (e.g. SYBR green).

RT-qPCR is a highly sensitive and specific method for detecting and quantifying nucleic acids that typically shows a dynamic range >6 logs. However, the properties of miRNAs present specific challenges for detection by RT-qPCR. The short length of miRNAs (21-23 nucleotides) does not provide sufficient ‘target space’ for amplification by conventional RT-qPCR, which requires forward and reverse primers that are both 18-20 nucleotides in length. Additionally, many miRNAs are members of families of transcripts with related sequences, some of which differ in only a single nucleotide [43] whereas the heterogeneity of miRNA processing means that only one miRNA species, generally the most abundant isoform, can be detected by a given RT-qPCR assay. To meet these challenges, RT-qPCR-based methods for miRNA detection have made use of either (a) gene-specific reverse transcription with a stem-loop primer followed by probe-based target detection (this method is the focus of this article, Figure 2a) [44], or (b) homopolymeric tailing of all miRNAs, reverse transcription with oligo dT primers followed by intercalating dye-based target detection (Figure 2b) [45,46]. Given that there is limited ‘target space’ when quantifying miRNAs, designing primer and probe sequences with optimal properties (specifically primer T_m_ = 60°C) may not be possible. As a result, primer overhangs or LNA-modified primer nucleotides have been utilized in order to increase primer T_m_[47]. Small RNA TaqMan assays can discriminate between two miRNAs with a single nucleotide mismatch [44] and are therefore specific for individual miRNA family members, but are unable to distinguish differentially processed isomiRs. Appropriate controls for RT-qPCR experiments include no template controls (NTCs) and Reverse Transcriptase minus controls (RT-) which monitor contamination of reagents with amplicon and genomic DNA background respectively. For miRNA detection, RT- controls are less important due to the manner in which the detection technologies work, which necessarily exclude amplification of background genomic DNA. Synthetic RNA oligonucleotides, or RNA extracted from cells or tissues known to express a particular miRNA of interest, can be used as positive controls. qPCR reactions are run in at least duplicate format. Replicates of RNA extraction (where sufficient material is available) and at the reverse transcription stage may also be used.

Upon completion of a qPCR run, the amplification curve data for each reaction are reduced to a single value (the quantification cycle, Cq). Cq is defined as the cycle at which the amplification curve crosses the threshold line. The threshold line is generally set automatically by the qPCR analysis software to a value several standard deviations above the background fluorescence, to a point at which the PCR reaction is in the log linear phase of amplification. Cq data can be exported from the qPCR analysis software in a spreadsheet format. For the purposes of relative quantification, gene-of-interest expression is normalized to a reference gene (often also called a housekeeping gene) that is assumed to exhibit stable expression between experimental groups in order to control for differential loading of template between reactions. Correct normalization is critical for accurately determining relative gene expression. Analysis using a highly variable reference gene will effectively scramble the gene-of-interest data, thereby reducing statistical power and obscuring biological meaning. Conversely, analysis using a reference gene that changes biologically between comparison groups can hide real changes between groups, and show changes where there are none. However, in the case of biofluids there are no clearly established reference miRNAs. Furthermore, we have recently shown that there is a tendency for serum from the dystrophin-deficient *mdx* mouse to have a higher miRNA and total RNA content than serum from wild-type non-dystrophic mice [42]. As a result, standardization of RNA input at the reverse transcriptase step, or normalization to an endogenous reference miRNA, will inevitably lead to quantification errors in this context (and likely in other cases). The ideal method of normalization for serum miRNAs is therefore to utilize an external spike-in control oligonucleotide added to each sample at the phenolic extraction phase. This method necessitates that RNA is extracted from a fixed volume of biofluid and samples processed equivalently. Having determined Cq values for both gene-of-interest and reference miRNAs, an arithmetic method is then used to compare between experimental samples. The Pfaffl method is a particularly useful approach as it takes into account the PCR efficiency of each assay [48] (Equation 1). To compute expression using the Pfaffl equation, Cq values and PCR efficiencies are required for the gene-of-interest and reference miRNA assays. In practice, PCR reactions are often less than 100% efficient, especially in the case of miRNA assays where possible primer sequences are very limited. Failure to account for PCR efficiency results in errors in the quantification of miRNA abundance (this error grows depending on the ΔCq such that larger fold changes between samples will be more exaggerated). If the PCR efficiency is unknown it can be approximated at 100% (E = 2), although determination of PCR efficiency and assay validation are strongly recommended. NOTE: When both the gene-of-interest and reference genes have E values equal to 2, the Pfaffl equation reduces to the commonly used Livak (ΔΔCq) equation [49].

(1) $$\mathrm{Expression}=\frac{{E_{\mathrm{GOI}}}^{-{\mathrm{Cq}}_{\mathrm{GOI}}}}{{E_{\mathrm{REF}}}^{-{\mathrm{Cq}}_{\mathrm{REF}}}}$$

E_GOI_ = PCR efficiency for gene-of-interest assay

E_REF_ = PCR efficiency for reference gene assay

Cq_GOI_ = Quantification cycle for gene-of-interest assay

Cq_REF_ = Quantification cycle for reference gene assay

PCR efficiency can be determined by applying linear regression analysis to the exponential phase of the amplification curve for each PCR reaction using a program such as LinRegPCR [50]. The RDML format (Real-time PCR Data Markup Language) is used to transfer data from the real-time PCR instrument to the LinRegPCR software [51]. In this approach, a window-of-linearity is determined for each amplification curve (i.e. the cycle vs log(fluorescence) plot) and a straight line fitted to this region. The PCR efficiency for each reaction can then be determined from the gradient of the fitted line as according to Equation 2. The average PCR efficiency for each assay over all reactions is then used in the Pfaffl analysis.

(2) $$E=10^{\mathrm{gradient}}$$

While relative quantification of serum miRNA levels is generally sufficient for the majority of research studies, absolute quantification has a number of advantages. The determination of miRNA copies per volume of serum allows for the direct comparison of individuals analyzed at different times and the comparison of the relative abundances of different miRNAs. Absolute quantification is achieved by analyzing a standard curve (i.e. cDNA samples generated from a dilution series of synthetic miRNA oligonucleotides) in parallel with experimental samples.

## Results and discussion

**Figure 3:**
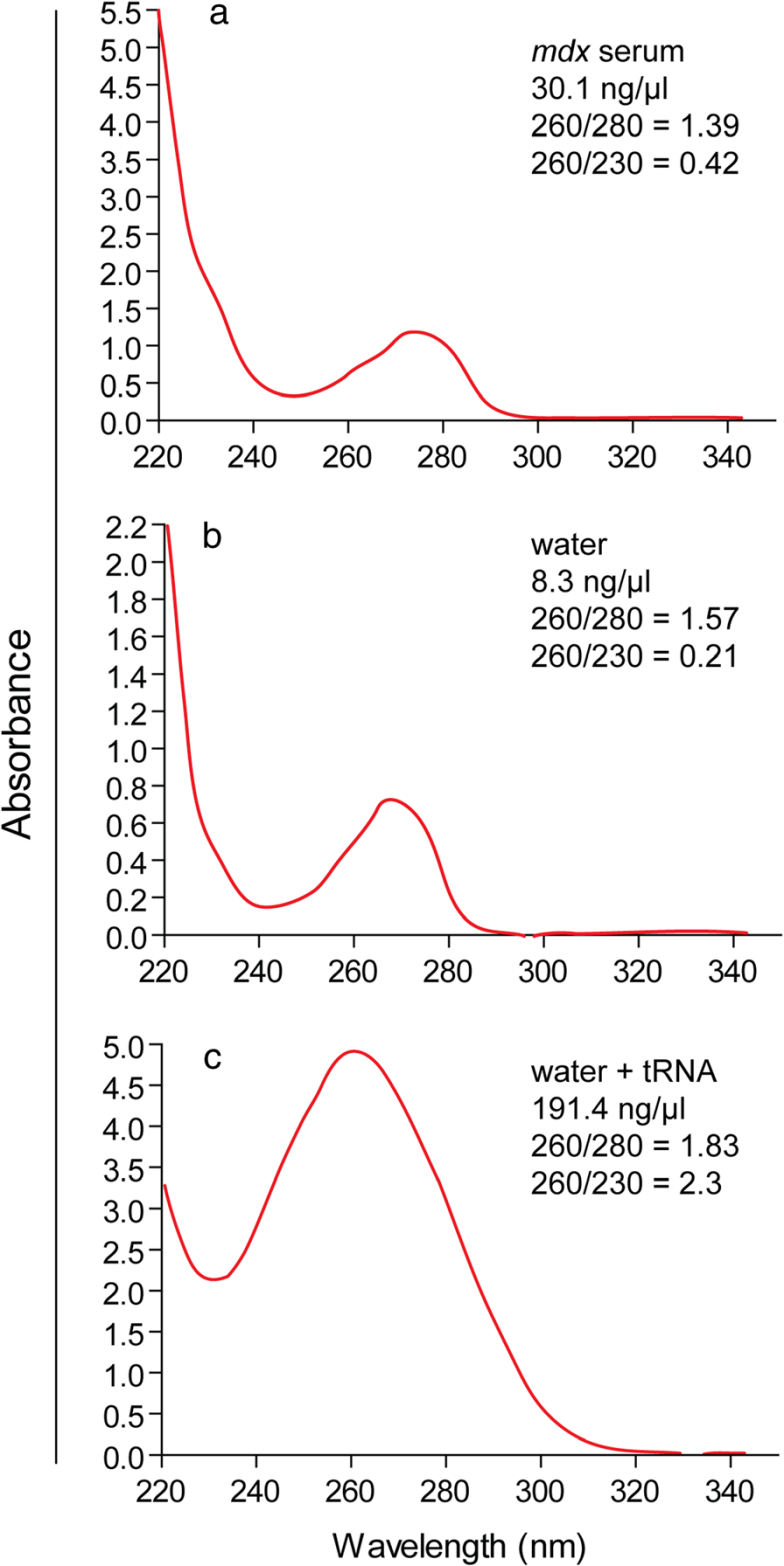
**Assessment of serum RNA quality.** Nanodrop traces for **(a)** an RNA sample extracted from *mdx* mouse serum, **(b)** a mock extraction from water, **(c)** yeast tRNA was added to the extracted water mock sample and re-analyzed.

The quality of RNA samples obtained from serum is generally poorer than for RNA extracted from cell cultures or tissues. As such, assessment of RNA concentration by 260 nm spectrophotometry (e.g. Nanodrop) is not possible [52]. Nanodrop traces of serum RNA samples exhibit features consistent with phenol contamination (i.e. a prominent absorbance peak at 270 nm which masks nucleic acid absorption peak at 260 nm; Figure 3a). A mock extraction in which the TRIzol LS protocol described below was performed on a water sample produced an identical trace (Figure 3b). Yeast tRNA was added to the mock extracted sample to a final concentration of ~200 ng/μl and re-analyzed. The resulting Nanodrop trace resembles that of pure RNA (Figure 3c) indicating that (a) the RNA content of serum is below the lower limit of detection for the Nanodrop, and (b) that the 230 nm and 270 nm peaks are likely low level contamination present following phenol-chloroform based extractions and not specific to serum samples. Alternatively, total RNA concentration can be determined by RiboGreen assay or Bioanalyzer.

**Figure 4:**
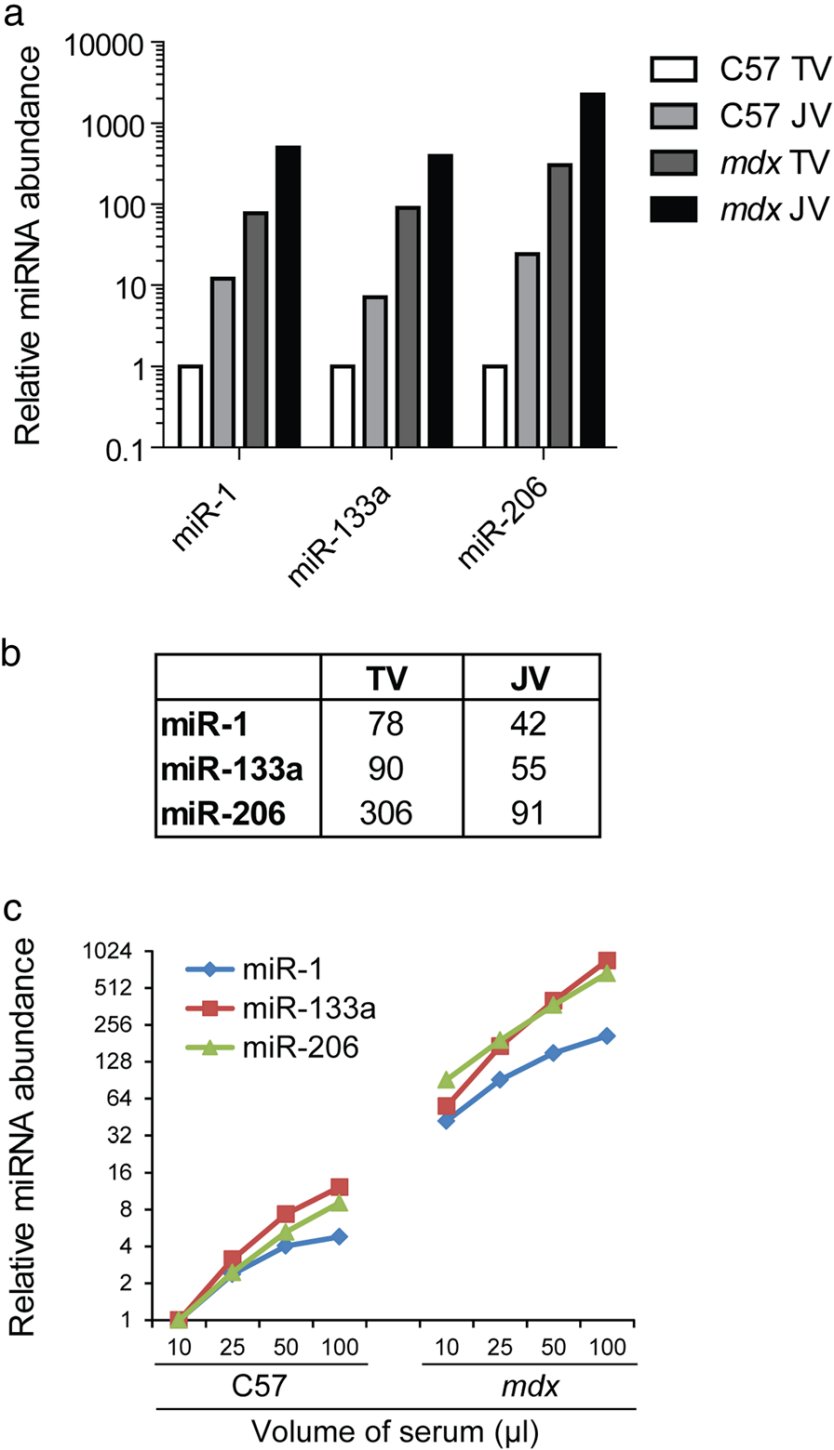
**miRNA quantification from different collection routes and biofluid volumes.** Whole blood was collected from the tail vein (TV) of a C57 (wild-type) mouse and an *mdx* (dystrophic) mouse. The mice were sacrificed and blood collected from the jugular vein (JV) from the same animals. Serum was prepared and RNA extracted. **(a)** Relative miRNA abundance was determined for each sample by small RNA TaqMan RT-qPCR for miR-1, miR-133a and miR-206 **(b)** Fold changes for *mdx* vs C57 for tail vein and jugular vein collections. **(c)** RNA was extracted from 10 μl, 25 μl, 50 μl and 100 μl of serum and relative miRNA abundance determined by small RNA TaqMan RT-qPCR for miR-1, miR-133a and miR-206.

Comparison of serum collection methods revealed that extracellular miRNAs were found to be ~10 times more concentrated in serum harvested from the jugular vein than for the tail vein for two different mouse strains (C57 and *mdx*) (Figure 4a). In the case of both extraction methods, miR-1, miR-133a and miR-206 were highly enriched in the *mdx* mouse (Figure 4b) as reported previously [28-33]. These data show that while both methods of blood harvesting are valid in their own right, the two types of extraction should not be compared within the same experiment. Similarly, to test the efficiency of RNA extraction from varying volumes of biofluid, serum was harvested from C57 and *mdx* mice, RNA extracted from 10 μl, 25 μl, 50 μl and 100 μl of serum, and miRNA abundance analyzed. A linear relationship was observed between the quantity of miRNA detected and the volume of serum analyzed for both strains as expected (Figure 4c), although miR-1 abundance plateaued in the 100 μl samples for both C57 and *mdx* mice. As a result, the optimal biofluid volume for analysis is 10-50 μl (subsequently made up to 200 μl with nuclease-free water).

**Figure 5:**
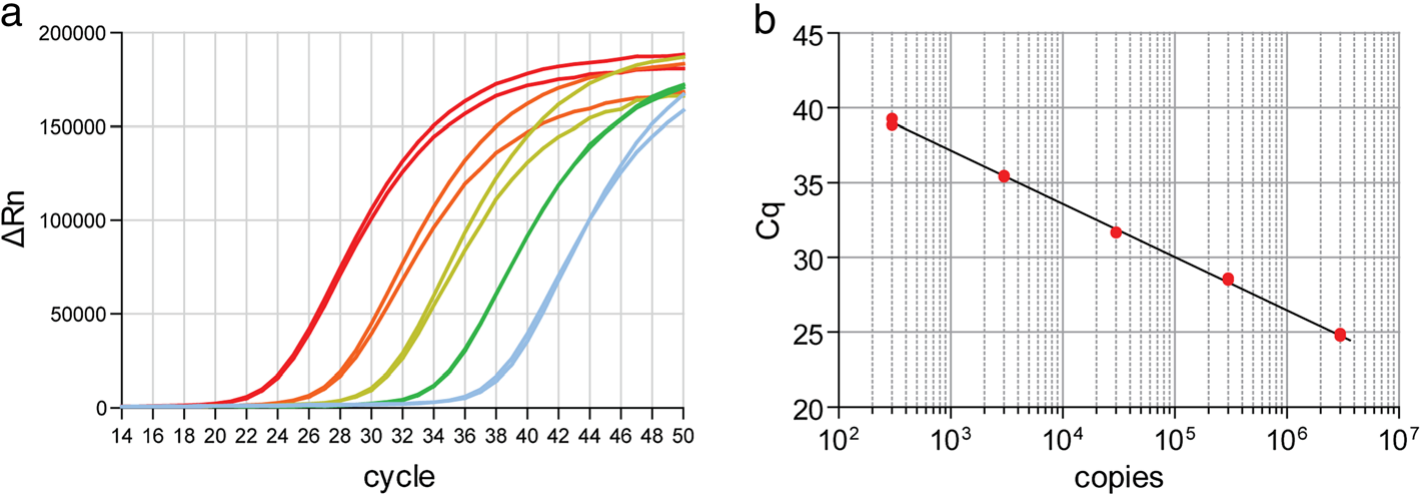
**Absolute quantification of synthetic miR-1 oligonucleotides.** A 10-fold dilution series of synthetic miR-1 oligonucleotides was diluted in 20 ng/μl yeast tRNA. RNA was extracted from each standard and reverse transcribed using stem-loop primers. The resulting cDNA was analysed by small RNA TaqMan RT-qPCR. The top standard contains 3 million copies and the bottom standard contains 300 copies. **(a)** Amplification plot. **(b)** Standard curve.

For absolute quantification, a 10 fold serial dilution series of a synthetic miR-1 oligonucleotide was prepared, reverse transcribed and analysed by qPCR. Linear amplification was observed across all standards covering a range of 3 million to 300 copies of miR-1 (5 logs) (Figure 5).

**Figure 6:**
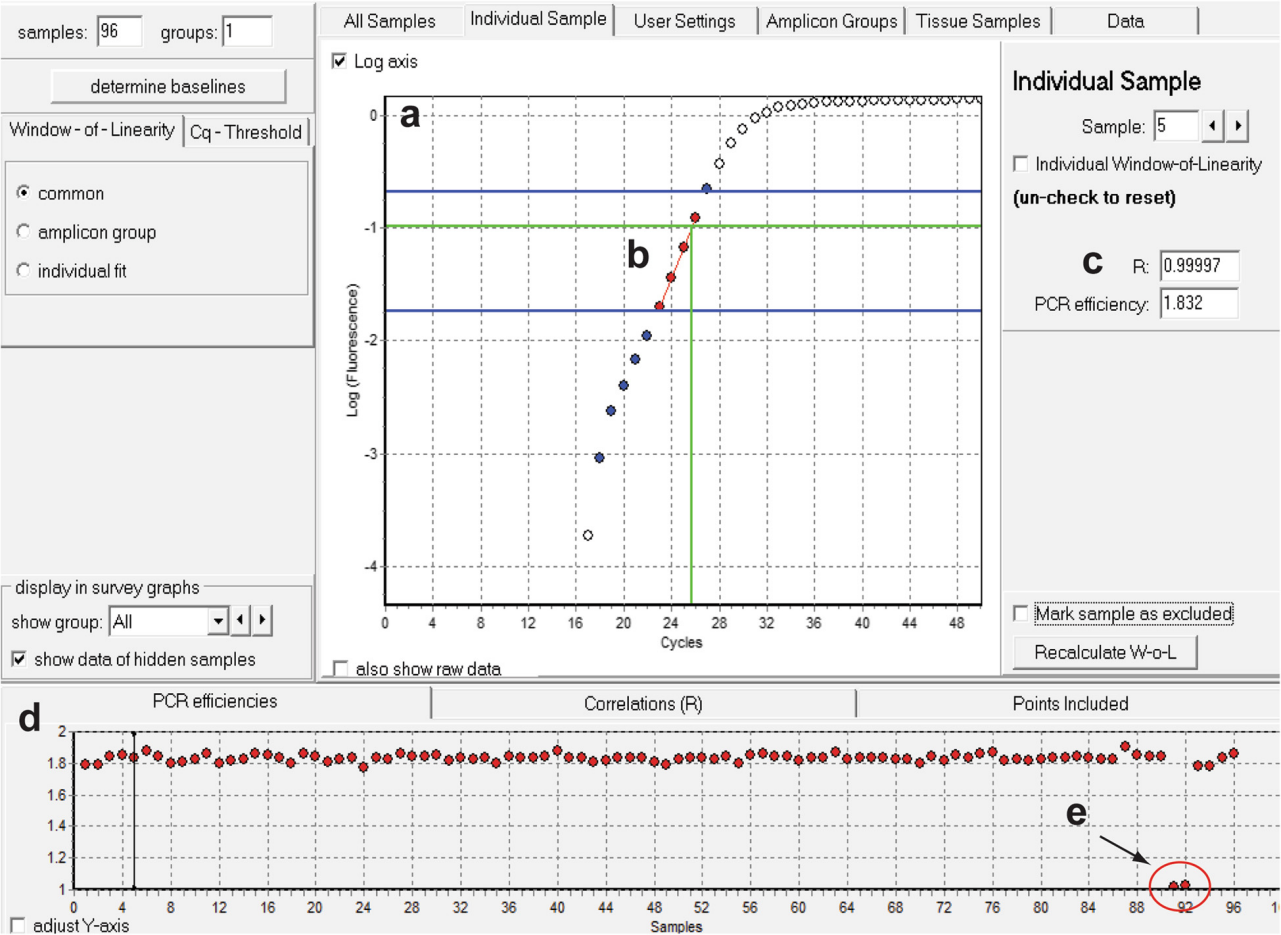
**Determination of PCR efficiency using LinRegPCR.** Screenshot of LinRegPCR software showing sample data with key feature highlighted. **(a)** Amplification plot (i.e. Log(Fluorescence) vs Cycle). **(b)** Window-of-linearity (included points are shown in red). **(c)** PCR efficiency and correlation coefficient (R) indicating how well the points fit the analysed linear region for a single reaction. **(d)** PCR efficiencies and correlation coefficients for all samples. **(e)** Two samples (the no template controls) failed to amplify and have PCR efficiencies ~1 (i.e. 0%).

**Figure 7:**
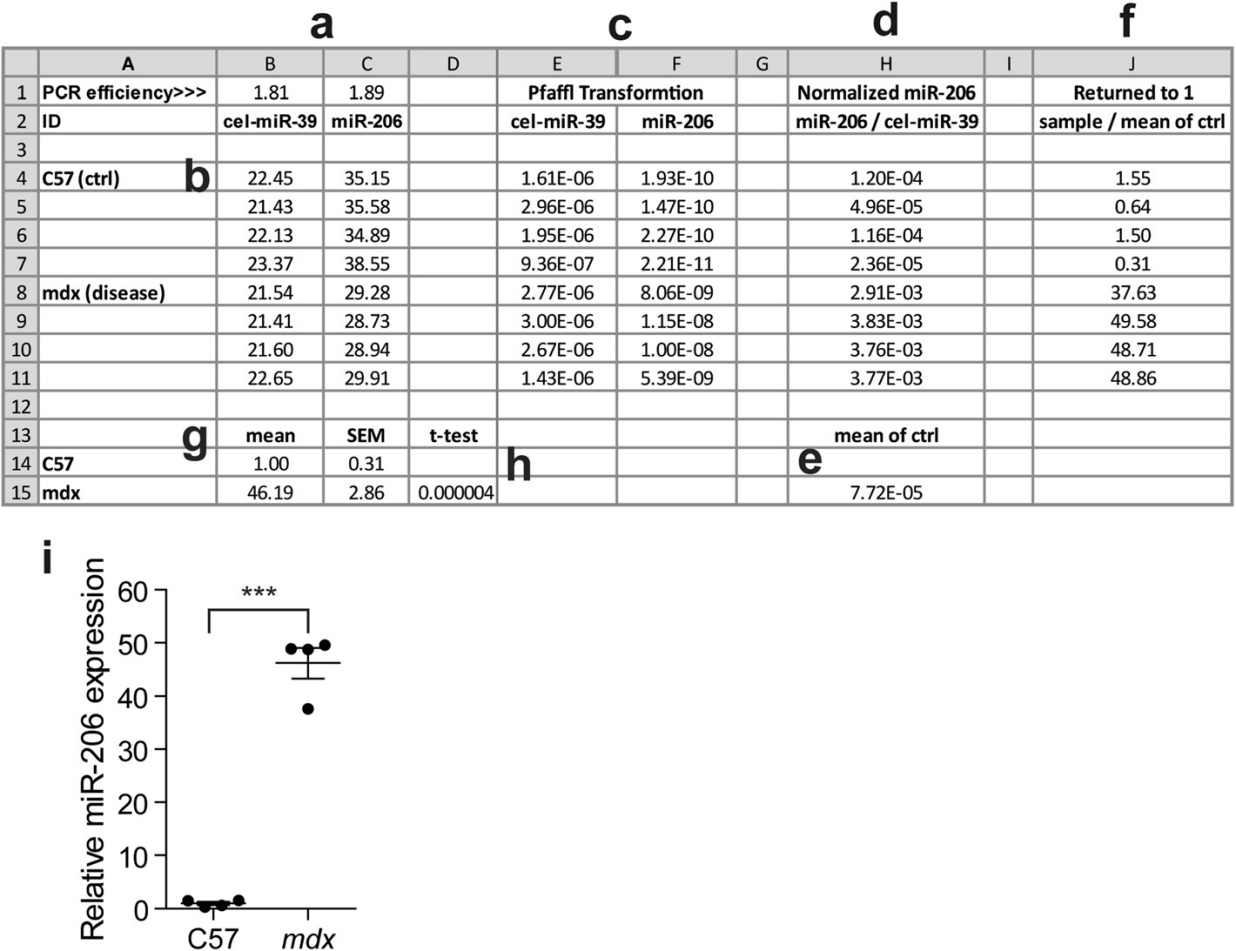
**Pfaffl analysis.** Example of Pfaffl calculation on serum miRNA RT-qPCR data performed in Microsoft Excel 2010. In this experiment, serum abundance of miR-206 was compared between C57 (wild type control) and *mdx* (dystrophic) mice aged 16 weeks (*n* = 4). **(a)** PCR efficiencies determined experimentally for each assay. If PCR efficiency is unknown it can be approximated as 2 (i.e. 100%). **(b)** Cq data from RT-qPCR run. **(c)** Pfaffl transformation converts Cq values into amounts in arbitrary units [equation in E4, =POWER($B$2,-B4)]. **(d)** Gene-of-interest (miR-206) abundance is normalized by dividing the Pfaffl transformed quantity by that of the reference control (cel-miR-39) (equation in H4, =F4/E4). **(e)** The mean normalized quantity of the control is calculated [equation in H15, =AVERAGE(H4-H7)]. **(f)** Dividing by the mean of the control group returns the control to a value of one so that fold changes between experimental groups can be visualized more easily (equation in J4, =H4/$H$15). **(g)** The mean and standard error of the mean (SEM) are calculated for the two experimental groups. **(h)** A t-test performed in Microsoft Excel indicates a highly statistically significant difference between the groups [equation in D15, =TTEST(J4-J7,J8-J-11,2,2)]. **(i)** Graph of serum miRNA abundance data. Values are mean +/- SEM, ***P < 0.001.

Serum from 16 week old C57 and *mdx* mice was collected, RNA extracted and miR-206 and cel-miR-39 abundance determined by RT-qPCR. LinRegPCR was used to determine PCR efficiencies for each reaction by linear regression analysis (Figure 6). Relative miRNA abundance was determined using the Pfaffl method and miR-206 abundance normalized to the external spike-in control oligonucleotide (cel-miR-39). A worked example of the Pfaffl method is shown in Figure 7a-h. (The PCR efficiencies determined for cel-miR-39 and miR-206 are 81% and 89% respectively). miR-206 abundance was found to be ~46 fold higher in the *mdx* mouse with replicates grouping closely (SEM = 2.86). The difference between groups was highly statistically significant (*P* = 0.000004, unpaired t-test, 2 tailed, *n* = 4) (Figure 7i).

## Conclusions

Here we describe a well validated complete work flow for harvesting murine biofluid, extracting biofluid RNA, detecting and quantifying miRNA expression by RT-qPCR, and analysis of raw and processed data. These protocols allow for the determination of relative and absolute miRNA quantification with high accuracy, specificity and sensitivity.

## Methods

### Materials

#### Reagents

• Mice (e.g. data presented here involves wild-type C57Bl/10 and dystrophic C57BL/10ScSn-Dmd^mdx^/J [*mdx*]) **CAUTION:** Experiments involving live rodents must conform to all relevant institutional and governmental regulations for animal handling and care. All animal procedures described here are permitted under PPL 30/2907 awarded to Professor Matthew J. A. Wood at the University of Oxford by the UK Home Office in accordance with UK law (Animals [Scientific Procedures] Act 1986).

• TRIzol LS Reagent (catalogue #: 10296-028 [Life Technologies]). **CAUTION:** Always work with TRIzol LS reagent in a fume hood. Always wear a lab coat, gloves and safety glasses.

• Chloroform (molecular biology grade) **CAUTION:** Always work with Chloroform in a fume hood. Always wear a lab coat, gloves and safety glasses.

• Isopropanol (molecular biology grade).

• 75% Ethanol wash (molecular biology grade).

• RNase-free glycogen (catalogue #: 10901393001 [Roche CA, USA]).

• Nuclease-free water (RNase-, DNAse- pyrogen-free) (catalogue #: AM9932 [Life Technologies]).

• Synthetic single-stranded RNA oligonucleotide for use as an exogenous spike-in control. This can be any sequence not found in the host organism. For analysis of human and mouse serum the *Caenorhabditis elegans* miRNA cel-miR-39 is typically used (5′-UCACCGGGUGUAAAUCAGCUUG). Alternatively a cocktail of synthetic small RNAs can also be used, in which case multiple oligonucleotides are required. (NOTE: 5′ phosphorylation of the RNA oligonucleotide is not required).

• MicroRNA Reverse Transcription Kit (catalogue #: 4366597 [Life Technologies]).

• TaqMan Gene Expression Mastermix (catalogue #: 4369510 [Life Technologies]).

• Small RNA TaqMan assay (medium/large scale) (Life Technologies). (Assay IDs: miR-1 002222, miR-133a 002246, miR-206 000510, cel-miR-39 000200). RT primers are supplied at 20X concentration with medium and large scale assays. This concentration is more convenient for RT multiplexing whereas the small scale assays contain 5X RT primers which is less convenient.

• Yeast tRNA (catalogue #: 10109495001 [Roche]). 

• MS2 Bacteriophage RNA (catalogue #: 10165948001 [Roche]).

#### Equipment

• Dissecting scissors

• Dissecting forceps

• Microcentrifuge

• Ice bucket

• Flat bottom mouse restrainer (or equivalent)

• Warming chamber

• 0.5 ml Terumo Insulin Needles 29G (Myjector U-100)

• Microvette® CB300 capillary serum Blood Collection tubes (Sarstedt, Leicester, UK). For serum, clot-activator tubes (catalogue #: 16.440) and for plasma, Potassium-EDTA tubes (catalogue #: 16.444)

• Refrigerated centrifuge

• Vortex mixer

• 1.7 ml microcentrifuge tubes

• 0.6 ml strip tubes

• Conventional PCR thermocycler

• Real-time PCR instrument. For example, StepOne Plus with analysis software (StepOne Plus v2.3) (catalogue #: 4376373 [Life Technologies]). NOTE: PCR consumables/plastics are specific to each real-time instrument.

• MicroAmp® Fast Optical 96-well Reaction Plate with Barcode, 0.1 ml (catalogue #: 4346906 [Life Technologies])

• MicroAmp® Optical Adhesive Film (catalogue #: 4311971 [Life Technologies])

• Plastic wedge/roller for sealing plates

• LinRegPCR software http://www.hartfaalcentrum.nl/php/generic_download_action.php?table=files&fileName=LinRegPCR.zip

• 7-zip software http://www.7-zip.org/download.html

• Microsoft Excel

#### Reagent Setup

##### Spike-in control oligonucleotide/Synthetic miRNA standard oligonucleotides

Prepare 5 nM stock solutions in nuclease-free water. Prepare aliquots to minimize repeat freeze-thawing and store at -80°C.

##### Yeast tRNA solution

Prepare a 20 ng/μl solution in nuclease-free water. Concentration can be altered to mimic biological samples. Yeast tRNA solution should be stored at -80°C.

##### Bacteriophage MS2 RNA solution

Prepare a 1 μg/μl solution in nuclease-free water, aliquot and store at -80°C.

### Protocol

#### Harvesting Murine Serum and Plasma (TIMING: 3 hours)

1) Biofluids can be collected in a variety of ways depending on experimental requirements. (Different collection tubes are required for serum and plasma samples).

A. Collection from the Jugular Vein

i. Prepare bucket containing ice for storage of biofluid collection tubes.

ii. Disassemble the collection tube and detach the cap ready for use.

iii. Sacrifice mouse by cervical dislocation. Immediately proceed to next step.

**CRITCAL STEP:** Work quickly for steps iv to vii to avoid problems due to clotting.

iv. Place mouse on its back and remove skin and fur from neck region. This is done by carefully pinching the skin using forceps and then cutting the skin with scissors.

v. If the jugular veins in the neck are not visible, pull back skin (and/or cut away fat tissue) to expose them.

vi. Nick blood vessel with scissors. Blood will quickly pool in the neck area.

vii. Use the capillary tube to collect blood by touching the tip against the pooling blood. The collection tubes fill through a combination of gravity flow and capillary action. (For a 30 g mouse 2-4 full tubes can typically be collected, although a single tube is more than enough for most analyses).

**CRITCAL STEP:** Angle the tube downwards to allow it to fill by gravity flow. Avoid pushing the tube into the blood pool as this will prevent the filling by capillary action. If required, apply gentle pressure to the mouse in order to maximize biofluid recovery.

B. Collection from the tail vein

**CAUTION:** Performing tail vein bleed on live mice is classified as a ‘procedure’ according to UK law and requires approval from the UK Home Office. Obtain legal approval from host government or institution before performing this protocol.

i. Set the warming chamber to 37-39°C and allow the equipment to acclimatize to this temperature.

ii. Once the chamber has heated sufficiently, place the mice in a cubicle for 5-10 minutes. The extremities of the mice such as the feet and tail will become pink and mice will appear subdued.

**CRITCAL STEP:** It is imperative that the operator works quickly and efficiently from this point, as it is the heat which causes the vein to dilate and allows the easier visualization of the vein.

iii. Remove the mouse from the chamber, place it in the restraining device and secure it by limiting the space in the restrainer. NOTE: the mouse may experience some distress so ensure that the mouse has enough space so that it does not injure itself.

iv. Disassemble the collection tube and detach the cap ready for use.

v. The artery in the tail runs dorsally and the veins are located to the right and left of this vessel. Locate a vein and apply a tourniquet to the tail above the area to be punctured using the middle and index finger. Straighten the lower end of the tail using the ring finger and thumb.

vi. Using a 29G needle, puncture the vein by sliding the shaft of the needle under the skin into the vein, bevel down. Slide the needle gently in and out 3 times to widen the puncture site.

vii. Remove the needle and position the collection tube so as to be ready to collect the blood.

viii. Release the tourniquet but secure and straighten the tail to minimize movement from the mouse.

ix. Collect approximately 20-150 μl of blood (dependent on legal stipulations and experimental requirements) by pressing the tip against the edge of the blood droplet.

x. If insufficient blood is attained, repeat steps vi-ix until the desired volume is collected.

xi. To stop the bleeding, press the tail for ~5 seconds or until bleeding stops.

**CAUTION:** Dispose of used insulin needles safely according to institutional procedures.

2) Cap collection tubes and store whole blood on ice.

3) Store serum tubes at 4°C for at least 1 hour. This allows the samples to clot and begins the separation of the cellular and liquid components of the blood.

4) Spin at 10,000 *g* for 5 minutes at room temperature using a bench top centrifuge.

5) Transfer the serum/plasma (supernatant) to a clean 1.7 ml microcentrifuge tube (pool serum samples derived from the same mouse. If any samples are visibly hemolysed [i.e. deep red colour], avoid pooling and store separately).

**CRITCAL STEP:** Be careful not to transfer any of the cellular blood as this will confound downstream analysis. ~50% of the collected volume is expected to be recovered as serum/plasma with the remainder being the cellular component.

**PAUSE POINT:** Store samples at -80°C until ready for RNA extraction.

#### RNA extraction from biofluids (TIMING: 3 hours)

The biofluid RNA extraction protocol detailed below is scalable, such that extracellular RNA can be extracted from any volume of biofluid. 200 μl is a convenient volume as it enables the entire protocol to be performed in 1.7 ml microcentrifuge tubes.

**CAUTION:** When working with RNA always use clean gloves, RNase-free plasticware and barrier pipette tips to prevent contamination of samples with RNases. Store RNA solutions on ice to minimize sample degradation.

6) Aliquot a user-defined volume of biofluid into a clean RNase-free 1.7 ml microcentrifuge tube. We typically use 50 μl although the protocol can be easily scaled to accommodate larger volumes if required. (We have measured miRNA abundance from as little as 10 μl of biofluid). Using a constant volume of biofluid between samples is highly recommended so that samples prepared from different experiments can be directly compared.

7) Adjust the volume of biofluid to 200 μl using nuclease-free water.

8) Immediately add 600 μl of TRIzol LS reagent and mix by vortexing for 10 seconds.

9) Add 3 μl of 5 nM external spike-in control to each sample and mix by vortexing for 10 seconds.

10) **OPTIONAL**: Add 1 μl of 1 μg/μl MS2 Bacteriophage carrier RNA solution to improve extraction efficiency [41].

11) Incubate TRIzol LS/biofluid mixture for 5 minutes at room temperature.

12) Add 160 μl of chloroform to each sample and mix by vortexing for 15 seconds.

13) Incubate TRIzol LS/chloroform mixture for 15 minutes at room temperature.

14) Centrifuge the samples at 12,000 *g* for 15 minutes at 4°C. After centrifugation the mixture separates into three phases. The clear aqueous top phase contains the RNA.

15) Transfer the aqueous phase to a clean 1.7 ml microcentrifuge tube.

16) **OPTIONAL**: Re-hydrate the phenolic phase by adding an additional volume of nuclease free water in order to maximize RNA recovery [5]. Vortex samples, repeat phase separation, pool the aqueous layers and proceed with isopropanol precipitation.

17) **CRITCAL STEP:** Add 1 μl RNase-free glycogen to each sample to ensure efficient RNA extraction.

18) Precipitate the RNA by adding 400 μl of isopropanol.

19) Mix samples by vortexing.

20) Incubate samples for 10 minutes at room temperature.

**PAUSE POINT:** Samples can be precipitated overnight if necessary.

21) Centrifuge at 12,000 *g* for 10 minutes at 4°C. The RNA precipitate forms a gel like pellet at the bottom of the tube.

22) Discard the supernatant.

23) Wash the RNA pellet with 1 ml of 75% ethanol.

24) Vortex to resuspend the pellet and centrifuge at 7,500 g for 5 minutes at 4°C.

25) Discard the ethanol wash.

26) Pulse-spin the samples using a bench-top microcentrifuge and remove the residual ethanol wash using a narrow pipette.

27) Discard the supernatant and air-dry pellet for 10 minutes at room temperature.

28) Dissolve the RNA pellet in 30 μl RNase-free water and mix by pipetting. NOTE: It is recommended to always use the same volume of water when resuspending RNA pellets so that samples harvested from different experiments can be compared directly.

29) Incubate for 10 minutes at 55°C.

**PAUSE POINT:** Store samples at -80°C until ready for reverse transcription.

#### Reverse transcription (TIMING: 2 hours)

30) Prepare the following reverse transcription cocktail for each sample and aliquot into separate 0.6 ml PCR tubes (Table 1). Ensure that at least 10% excess cocktail is prepared to account for losses during pipetting. RT reactions are multiplexed so that multiple miRNAs can be reverse transcribed in the same reaction.

31) Add 5 μl of biofluid RNA sample to each aliquot of reverse transcription cocktail.

32) Mix by gentle pipetting and pulse spin to collect liquid at the bottom of the tubes.

33) Incubate reactions at 4°C for 5 minutes.

34) Incubate sample tubes in a PCR thermocycler and incubate as shown in Table 2:

**PAUSE POINT:** Store cDNA at -20°C.

**Table 1:** RT reaction components

**Component**	**μl per reaction (1X)**
10X miRNA RT buffer	2
MultiScribe RT (50 U/μl)	1
100 mM dNTP mix	0.15
RNase Inhibitor (20 U/μl)	0.18
20X miRNA RT primer - assay A	1
20X miRNA RT primer - assay B	1
20X miRNA RT primer - assay C	1
20X miRNA RT primer - assay D	1
Water	7.67
(Total)	(15)

**Table 2:** RT reaction conditions

**Time (minutes)**	**Temperature (°C)**
30	16
30	42
5	85
∞	4

#### Quantitative polymerase chain reaction (TIMING: 3 hours)

**CAUTION:** Prevent PCR amplicon carryover during preparation of qPCR by using clean gloves, barrier pipette tips, assign dedicated pre- and post-PCR areas and avoid opening plates after run completion.

35) Prepare the following RT-qPCR cocktail as shown in Table 3 for each sample or standard. Ensure that at least 10% excess cocktail is prepared to account for losses during pipetting.

36) Pipette the RT-qPCR cocktails onto the MicroAmp® Fast Optical 96-well Reaction Plate.

37) If a standard curve is required, prepare appropriate RT-qPCR cocktails (see steps 51-52).

38) Apply a MicroAmp® Optical Adhesive Film to plate and seal the wells using a plastic wedge or roller.

39) Spin the plate at 1,000 *g* for 1 minute to collect the liquid at the bottom of the plate wells.

40) Define the plate layout and reaction cycling conditions in the real-time software as shown in Table 4:

41) Place the sealed plate in the real-time instrument and initiate the qPCR run.

42) When the run has finished proceed with data analysis.

**Table 3:** qPCR reaction components

**Component**	**μl per reaction (1X)**
TaqMan small RNA assay (20X)	1
Product from RT reaction/standard	1.33
TaqMan mastermix	10
Nuclease-free water	7.67
(Total)	(20)

**Table 4:** qPCR reaction conditions

**Time**	**Temperature (°C)**
10 minutes	95
Then 40 cycles of:	
15 seconds	95
1 minute	60 (collect data)

**PAUSE POINT:** Data analysis can be performed at any time following acquisition of Cq data following completion of the qPCR run.

#### Analysis of raw data (TIMING: <1 hour)

43) Inspect data to ensure that all reactions show amplification curves as expected. (Sigmoidal or parabolic amplification curves indicate that fluorescence background is set incorrectly and can be adjusted manually).

44) Check that negligible amplification is observed in negative controls (i.e. NTC and RT-).

45) If intercalating dye methods (i.e. SYBR green) were used, check all reactions for expected melting profiles. Any reactions with multiple melt peaks or atypical melt profiles should be excluded from the analysis.

46) Ensure that the y-axis for the amplification plot display is set to log scale and adjust the threshold value so that it is within the log linear phase of amplification across all reactions. (It is highly recommended that the threshold value for each assay be standardized across plates as this enables between plate comparisons to be made).

47) If a standard curve was prepared, check that the estimated PCR efficiency is between 80% and 110%.

48) If a standard curve was prepared, ensure that all samples fall within the linear range of the assay. Any samples that amplify outside of the range of the standard curve should be flagged as ‘Out-of-Range’. In particular, reactions that amplify after the lowest standard should be considered ‘Not Quantified’ or ‘Not Detected’ as appropriate.

49) If a serial dilution series of template cDNA was used to prepare the standard curve, check that template inhibition does not occur in the high standard reactions. If template inhibition is observed, any samples that amplify in the problematic range should be excluded from the analysis.

50) Export data from the analysis software to Microsoft Excel or an equivalent spreadsheet software package. Exported data include Cq values, amount in arbitrary units or amount in copies for relative quantification (Pfaffl analysis), relative standard curve and absolute standard curve analysis methods respectively.

#### Final data analysis (TIMING: <1 hour)

51) Determination of PCR efficiency.

A. LinRegPCR.

i. Export RDML file from qPCR software.

ii. Change file extension from **.rdml** to **.zip** (ensure windows is set to view file extensions).

iii. ‘Extract all’ from the new **.zip** file.

iv. In extracted folder change the name of the file **rdml_data.xml** to **my_experiment.xml**.

v. Re-zip the **my_experiment.xml** file to **.zip** format using the 7-zip software.

vi. Rename my_experiment.zip to **my_experiment.rdml**.

vii. Open LinRegPCR software.

viii. Click ‘File, Read from RDML’.

ix. Select ‘Hydrolysis Probe’ in the ‘Monitoring Chemistry’ menu.

x. Select ‘ss cDNA’ from the ‘Amplification of’ menu.

xi. Select ‘cDNA sequence’ from the ‘Probe is targeting’ menu.

xii. Select ‘No’ from the ‘Data are baseline corrected’ menu.

xiii. Click on ‘Open RDML File’ button and browse to the location of **the my_experiment.rdml** file.

xiv. Click ‘OK’ to load the data.

xv. Click ‘determine baselines’ to initiate analysis.

xvi. When analysis is complete, click ‘File, Save to Excel’ (Microsoft Excel must be open).

xvii. Click ‘OK’.

xviii. The PCR efficiency for each individual reaction analysed is shown in column B of the Excel worksheet.

xix. The mean PCR efficiency for the analysed plate is shown in column G of the Excel worksheet. These values are utilized in the Pfaffl analysis.

B. cDNA Standard Curve

i. Prepare a serial dilution (2-10 fold as required) of RT reaction product (from step 34).

ii. Perform qPCR as described in steps 35-42. Assign relevant reactions as standards in the qPCR software as appropriate.

iii. An estimate of PCR efficiency is generally given automatically by the qPCR software. Alternatively, a standard curve can be drawn manually by plotting Cq values against log_10_(cDNA amount) for the serially diluted samples. PCR efficiency ‘E’ can be estimated from the gradient of the standard curve using Equation 3. A gradient of -3.32 corresponds with 100% PCR efficiency (E = 2).

(3) $$E=10^{\frac{-1}{\mathrm{gradient}}}$$

C. Absolute quantification

i. Estimates of PCR efficiency are not required for absolute quantification.

52) miRNA quantification

A. Pfaffl analysis

i. Pfaffl analysis is performed on Cq values exported from step 50 and requires PCR efficiency values determined in step 51. The Pfaffl equation is shown in Equation 1.

ii. For each reaction, determine the amount of starting material by raising the value of the PCR efficiency to the power of –Cq.

iii. Divide the gene-of-interest values by the reference values to obtain normalized values.

iv. Scale the data by returning one of the experimental groups to a value of 1. i.e. divide each normalized value by the mean normalized value for the control group.

B. Absolute quantification

i. Prepare a standard curve of synthetic RNA oligonucleotides as follows:

ii. Prepare 5 nM stocks of each synthetic oligonucleotide as described above (3.01 × 10^9^ miRNA copies/μl).

iii. Add 3 μl of each 5 nM oligonucleotide to a fresh 1.7 ml microcentrifuge tube.

iv. Make up to 1 ml using nuclease free water [or 20 ng/μl yeast tRNA solution as required] (9.03 × 10^6^ miRNA copies/μl).

v. Prepare 10 fold dilution series (with 5 steps) using 20 ng/μl yeast tRNA solution as a diluent.

vi. Use 5 μl of oligonucleotide/tRNA solution per 20 μl reverse transcriptase reaction.

vii. Use 1.33 μl of cDNA per 20 μl qPCR reaction. (For the top standard, each qPCR reaction contains 3 million copies of synthetic target oligonucleotide).

viii. Reverse transcribe standards as described in steps 30-34 and perform qPCR as described in steps 35-42.

ix. Copies per sample are determined by comparing samples against the standard curve.

### Troubleshooting

Troubleshooting advice can be found in Table 5.

**Table 5:** Troubleshooting advice

**Step**	**Problem**	**Solution**
1-5	Insufficient volume collected	Biofluid must be collected instantly post-mortem to avoid clotting related problems. The angle of the collection tube is critical. Avoid getting blood on the outside of the collection tube as the surface tension will allow the sample to flow back out of the tube.
	Hemolysed sample	Avoid vigorously mixing or shaking blood collection tubes. Minimize contact of blood with water or exposure to heat and ensure that samples are kept upright. Do not freeze blood samples before separation.
6-29	Low RNA yield	RNA yield is expected to be very low (~0.1 ng/lμl). Improved yield may be obtained by increasing biofluid volume (scale reagents accordingly). Increasing precipitation time and including the optional re-hydration of phenol phase and MS2 RNA carrier steps can also improve RNA recovery. Re-suspending the RNA pellet in a smaller volume increases concentration but may decrease yield.
43-50	No amplification	May be a real result. Include a positive control to verify.
	Atypical amplification curve	Adjust fluorescent background. If the shape of the curve does not improve exclude effected wells.
	Atypical melt profile (if applicable)	Exclude wells. Melting artefacts may occurs at low copy numbers or be caused by sub-optimal assay design. Switch to a probe-based detection technology or re-design assay.
	Template inhibition	Exclude samples over the range where inhibition is observed. Dilute samples (and therefore also PCR inhibitors) and re-run qPCR.
	Amplification in NTC or RT- reactions	Indicates reagent contamination. Switch to new reagents and clean work surfaces.
	Low PCR efficiency	Some assays may exhibit sub-optimal PCR efficiencies due to restricted target space. Improvements can be obtained by optimizing annealing temperature in the PCR protocol. Sub-optimal PCR efficiencies can be used with caution. Consider re-designing assay or switching to an alternative detection technology.

### Timing

Steps 1-5 Harvesting murine serum and plasma: 3 hours.

Steps 6-29 RNA extraction from biofluids: 3 hours.

Steps 30-34 Reverse transcription: 2 hours.

Steps 35-42 qPCR: 3 hours.

Steps 43-50 Analysis of raw data: <1 hour.

Steps 51-52 Final data analysis : <1 hour.

## Abbreviations

Cq: Quantification cycle; CSF: Cerebral spinal fluid; DMD: Duchenne muscular dystrophy; miRNA: microRNA; NTC: No template control; PCR: Polymerase chain reaction; RDML: Real-time data markup language; RT-: No RT control; RT-qPCR: Reverse Transcriptase-quantitative Polymerase Chain Reaction; siRNA: Small interfering RNA; Tm: Melting temperature.

## Competing interests

The authors declare no competing financial interests.

## Authors’ contributions

TCR contributed the majority of the protocol. CB contributed the tail vein blood collection protocol. All authors contributed data. TCR wrote the initial draft and all authors contributed to the final draft. All authors read and approved the final manuscript.
